# Effectiveness of a Multifaceted Mobile Health Intervention (Multi-Aid-Package) in Medication Adherence and Treatment Outcomes Among Patients With Hypertension in a Low- to Middle-Income Country: Randomized Controlled Trial

**DOI:** 10.2196/50248

**Published:** 2024-06-19

**Authors:** Muhammad Arshed, Aidalina Mahmud, Halimatus Sakdiah Minhat, Poh Ying Lim, Rubeena Zakar

**Affiliations:** 1 Department of Community Health Faculty of Medicine and Health Sciences Universiti Putra Malaysia Serdang Malaysia; 2 University Institute of Public Health, Faculty of Allied Health Sciences, University of Lahore, Punjab Lahore Pakistan; 3 Department of Public Health Institute of Social and Cultural Studies University of the Punjab Lahore Pakistan

**Keywords:** mobile health, mHealth, intervention, medication adherence, hypertension, low- to middle-income country, effectiveness, randomized controlled trial, Pakistan, drug adherence, tool, mHealth module, self-efficacy, systolic blood pressure, feedback

## Abstract

**Background:**

The high prevalence of uncontrolled hypertension in Pakistan is predominantly attributed to poor medication adherence. As more than 137 million people in Pakistan use cell phones, a suitable mobile health (mHealth) intervention can be an effective tool to overcome poor medication adherence.

**Objective:**

We sought to determine whether a novel mHealth intervention is useful in enhancing antihypertensive therapy adherence and treatment outcomes among patients with hypertension in a low- to middle-income country.

**Methods:**

A 6-month parallel, single-blinded, superiority randomized controlled trial recruited 439 patients with hypertension with poor adherence to antihypertensive therapy and access to smartphones. An innovative, multifaceted mHealth intervention (Multi-Aid-Package), based on the Health Belief Model and containing reminders (written, audio, visual), infographics, video clips, educational content, and 24/7 individual support, was developed for the intervention group; the control group received standard care. The primary outcome was self-reported medication adherence measured using the Self-Efficacy for Appropriate Medication Adherence Scale (SEAMS) and pill counting; the secondary outcome was systolic blood pressure (SBP) change. Both outcomes were evaluated at baseline and 6 months. Technology acceptance feedback was also assessed at the end of the study. A generalized estimating equation was used to control the covariates associated with the probability of affecting adherence to antihypertensive medication.

**Results:**

Of 439 participants, 423 (96.4%) completed the study. At 6 months post intervention, the median SEAMS score was statistically significantly higher in the intervention group compared to the controls (median 32, IQR 11 vs median 21, IQR 6; U=10,490, *P*<.001). Within the intervention group, there was an increase in the median SEAMS score by 12.5 points between baseline and 6 months (median 19.5, IQR 5 vs median 32, IQR 11; *P*<.001). Results of the pill-counting method showed an increase in adherent patients in the intervention group compared to the controls (83/220, 37.2% vs 2/219, 0.9%; *P*<.001), as well as within the intervention group (difference of n=83, 37.2% of patients, baseline vs 6 months; *P*<.001). There was a statistically significant difference in the SBP of 7 mmHg between the intervention and control groups (*P*<.001) at 6 months, a 4 mmHg reduction (*P*<.001) within the intervention group, and a 3 mmHg increase (*P*=.314) within the controls. Overall, the number of patients with uncontrolled hypertension decreased by 46 in the intervention group (baseline vs 6 months), but the control group remained unchanged. The variables groups (adjusted odds ratio [AOR] 1.714, 95% CI 2.387-3.825), time (AOR 1.837, 95% CI 1.625-2.754), and age (AOR 1.618, 95% CI 0.225-1.699) significantly contributed (*P*<.001) to medication adherence. Multi-Aid-Package received a 94.8% acceptability score.

**Conclusions:**

The novel Multi-Aid-Package is an effective mHealth intervention for enhancing medication adherence and treatment outcomes among patients with hypertension in a low- to middle-income country.

**Trial Registration:**

ClinicalTrials.gov NCT04577157; https://clinicaltrials.gov/study/NCT04577157

## Introduction

Hypertension is a significant health challenge worldwide and a leading cause of morbidity and mortality [1]. In the 21st century, hypertension has become a growing health issue worldwide. It is expected to increase from 918 million individuals in 2000 to 1.56 billion in 2025 [1]. Compared to high-income countries, the prevalence of hypertension among adults is more remarkable in low- and middle-income countries (LMICs) [2,3]. Hypertension is responsible for approximately 9.4 million fatalities worldwide, making it a significant cause of death [4]. These deaths are mostly preventable, as lowering the systolic blood pressure (SBP) can lessen fatalities from all causes and cardiovascular disease (CVD) [5].

The risk of mortality due to cardiovascular events and stroke is lowered by twofold for each 20 mmHg drop in the SBP or each 10 mmHg drop in the diastolic blood pressure (DBP) between the ages of 40 and 69 years [6]. The dosage of blood pressure medications administered and adherence to therapy are 2 important aspects that influence blood pressure control in patients receiving treatment with clinically corrected blood pressure levels. Patient compliance is a critical aspect of blood pressure management, and medication is worthless for those who refuse to take it [7]. What is more worrying is the chronic nature of hypertension and the need to be compliant with the medications usually for more than 1 year. It has been noted that 1 year after starting antihypertensive medication, 50% of patients still use it [8,9]. Unfortunately, in general, the percentage of treated patients with hypertension who achieve control levels is only between 20% and 50% [2,10].

In Pakistan, hypertension is a crucial matter of public health, where nearly 19% of the youth and 33% of individuals older than 45 years have hypertension, with the majority of the population with hypertension having poor blood pressure control [11]. Poor medication adherence has been noted to contribute to poor blood pressure control in Pakistan [12]. A recent investigation found that 37.7% of patients fail to take their antihypertensive medications, as directed [12]. This situation is of concern because as mentioned, medication adherence is a proven and cost-effective treatment for hypertension [13], in addition to lifestyle modification and medical risk assessment. Furthermore, medications may lower the risk of stroke and myocardial infarction by 30% and 15%, respectively, among the population with hypertension [14]. Lower levels of adherence are connected to poorer blood pressure control and unfavorable outcomes [15].

For a few years, there has been an upsurge in the use of mobile health (mHealth) apps to improve medication compliance [16,17]. Using mobile technology, such as cell phones, personal digital assistants, patient-monitoring equipment, and other wireless devices, for medical care is referred to as “mHealth” [18]. mHealth is an ideal tool for LMICs due to its low cost and ease of use. For mHealth, all that is required are mobile devices, cellular communication technologies, and an internet connection. According to the Pakistan Telecommunication Authority, more than 137 million Pakistanis use cell phones, corresponding to a cellular density of 77% of the population [19]. However, despite the growing popularity of cell phones in LMICs, mHealth approaches in these countries remain limited.

Furthermore, no specific association between the use of mHealth apps and enhancement of medication adherence in CVD in LMICs has been shown to date [20]. Several studies have suggested that further investigations be conducted to determine whether mHealth can enhance medication adherence in LMICs [20,21] compared to traditional methods [22]. WhatsApp facilitates the collection of real-time data over both time and place. WhatsApp offers a plethora of health-related uses, including optimizing communication and the delivery of health education [23,24]. A survey found that Pakistanis primarily use social media for communication and information exchange in the health sector, with WhatsApp and YouTube being the most widely used social media platforms for health-related topics [25]. Several important observations, particularly those gleaned from the body of the existing literature [25], guide our decision to use WhatsApp in implementing this cutting-edge intervention, since it is an efficient way to provide interventions with respect to cost, time, and dissemination.

Using the Health Belief Model [26] and self-determination theory [27] as a foundation, we developed a novel mHealth intervention module for the population with hypertension in LMICs, particularly Pakistan. The module is called “Multi-Aid-Package.” It is a multifaceted intervention integrated with educational guidelines and a reminder component. The module addresses individual patients’ perspectives and concerns and incites health-related beliefs toward better medication adherence. This trial’s distinctive feature was its all-encompassing, multimodal strategy, which combined various previous interventions [28-30] into a single intervention. Second, animated images and videos were used in place of text in this study. Therefore, as far as we are aware, this is the first study in Pakistan to develop and assess the efficacy of a comprehensive and multifaceted mHealth intervention.

Consequently, this study sought to use the mHealth-based multifaceted intervention with the aid of WhatsApp to help patients who were not adhering to their medication and to assess the efficiency of Multi-Aid-Package in optimizing adherence to medication and manage the SBP among patients with hypertension in the LMIC context. We hypothesized that this mHealth module intervention would improve medication adherence, lower the SBP, and eventually lower the mortality and morbidity due to hypertension.

## Methods

### Trial Design

This trial was a parallel, single-blinded, superiority randomized controlled study that lasted for 6 months and had a 2-arm, parallel design. The trial was carried out following the CONSORT (Consolidated Standards of Reporting Trails) Statement 2010 standards [31]. Participants were randomly concurrently allocated 1 of 2 groups (intervention or control) in a 1:1 ratio. The intervention group underwent the Multi-Aid-Package intervention, while the control group received regular treatment (as per the hospitals’ routine practice) [32]. Evaluations were carried out at baseline and 6 months after the implementation of the intervention. The trial was registered with Clinical Trials (NCT04577157; registration date October 6, 2020) before the start of recruitment, which began on January 3, 2021.

### Sampling Method and Study Setting

A 2-stage random sample procedure was used to carry out sampling. The first stage required selecting a hospital at random from a list of hospitals, and the second stage entailed randomly selecting patients with hypertension from the selected hospital.

The study site selected was a public tertiary care hospital in Punjab’s provincial capital, Lahore. Lahore is Pakistan’s second-biggest city and has a population of 11,302,285, with a gross domestic product (GDP) of US $84 billion [33].

### Study Participants

Study participants were selected from among patients diagnosed with hypertension at the hospital’s cardiology and medical outpatient departments. Patient screening involved identifying those who were registered as hypertensive for the past month. The selection process was conducted by specially assigned registrars who screened patients using the Self-Efficacy for Appropriate Medication Scale (SEAMS) [34] and by asking the patients the number of pills they consumed during a specified period [35]. Although these 2 approaches to measure medication adherence are distinct, both adherence measures were used in this trial for inclusion/exclusion considerations. Only those patients who satisfied both criteria were recruited. Due to the selection criteria’s restriction to using both approaches, any participant could be classified as nonadherent. These results indicated the patients’ medication adherence status. Based on these assessments and other eligibility criteria, 439 participants were selected. The data collected included sociodemographic information, health-related profiles, baseline SEAMS score, and number of pills (representing medication adherence status). All the information was collected through face-to-face interviews conducted by trained research staff. In addition, each participant’s baseline SBP reading was also recorded.

### Eligibility Criteria

The inclusion criteria were as follows: patients who were at least 18 years old, diagnosed with hypertension within the previous month, prescribed antihypertensive medications, had poor medication adherence (a low SEAMS score ranging from 13-21 and pill-counting rate<80% were coded as nonadherent), had a smartphone with WhatsApp installed, and had the ability to read and send messages using WhatsApp.

The exclusion criteria were as follows: patients who had plans to leave the study area during the study period that would prohibit them from accessing cell signals; had a history of cancer, as they would need medication adjustments over time; would undergo a planned surgery or intervention; had blood pressure>220/120 mmHg (in the hypertensive emergency category); or were pregnant, breastfeeding, or 3 months postpartum.

### Sample Size

The sample size was estimated to assess a 1-point difference in SD (SD 2) on the major outcome metric of adherence change when comparing the 2 groups. To evaluate the 2-tailed hypothesis, the α level (type 1 error) was set at .05, with a 95% CI interval, Z=1.96, and strength to obtain a power of 90% [36]. The adherence reference value was as determined by a recent study [17]. After a 30% attrition rate, using Lemeshow et al’s [37] formula:

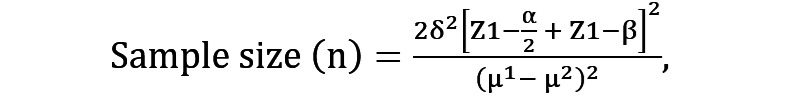



the calculated sample size for this study was 440 participants, equally allocated to the intervention and control groups (n=220, 50%, per group).

### Randomization and Concealment

A simple complete randomization method was used [38]. First, a random sequence was generated in Microsoft Excel using the formula =ROUNDUP (RAND ()*440,0). Participants were then split into 1 of 2 groups at random in a 1:1 ratio using their unique identification numbers. Opaque envelopes were used to disseminate information concerning participant allocation.

An independent biostatistician performed all the subsequent randomization steps. In addition, the staff involved in the randomization assessment and intervention delivery were separated, thus ensuring they did not know which patient belonged to which group.

### Blinding

The research team, which consisted of the research supervisor and research assistants responsible for data collection, was unaware of the intervention and control groups [39]. Due to the subjective nature of the intervention, participants were aware of their allocation to either the intervention or the control group.

### Outcome Measures

The primary outcome was the change in antihypertensive medication adherence at 6 months. This change was measured using the SEAMS questionnaire and self-reported pill counting (number of pills consumed over a certain period divided by pills prescribed for that specific period) [35]. SEAMS is a validated and reliable questionnaire, a 13-item assessment of medication self-efficacy in managing chronic conditions, found appropriate for individuals with limited literacy [34]. SEAMS uses a 3-point answer scale, where 1 denotes a lack of confidence, 2 denotes a moderate level of confidence, and 3 denotes a high level of confidence. A conceivable score is 13-39 points. Greater medication adherence is associated with higher scores, and vice versa. Based on prior studies, a cutoff value of 80% was used to distinguish between adherence status and nonadherence status. Participants were questioned regarding the number of pills they had been prescribed for a certain period, the number of pills they had consumed, and the number of pills they had forgotten to take during that certain period. Adherence rates were then calculated [35]. Patients who scored <80% were categorized as nonadherents, while those who scored ≥80% were classified as adherents [40]. At baseline, all participants were nonadherent; therefore, no further analysis could be conducted.

The secondary outcome was the SBP change at 6 months. This outcome was assessed in the hospital by a nurse who was not aware of the allocation of the study participants. The blood pressure was measured using a calibrated upper-arm mercury sphygmomanometer (MODEL-605P YAMASU). Standard principles were used to measure each participant’s blood pressure [41].

### Interim Analysis

At baseline, 3 months, and 6 months, both primary and secondary outcomes were evaluated. The 3-month analysis was used as a bridge to evaluate attrition rate patterns and the trends of change in outcomes. It was performed as an interim analysis; therefore, its results were not reported. The 6-month analysis was regarded as final.

### Intervention

The intervention’s main objective was to enhance adherence to antihypertensive therapy in the intervention group using Multi-Aid-Package, a novel mHealth module. Multiple procedures were used in the intervention development. The first step was a thorough literature search for the theories and determinants of medication nonadherence. Additionally, hypotheses on patient acceptance of electronic/mobile devices were looked up in the accessible literature. The procedure for consulting with a group of specialists came next. Experts in epidemiology, behavioral intervention, health education, IT, and cardiology specializing in hypertension management used the Health Belief Model, self-determination theory, and relevant clinical standards and recommendations in the development of this module.

The content of this module included 7 items, a multifaceted approach with educational instructions, and reminders. Multi-Aid-Package comprised written and voice reminders, as well as graphics-based reminders (GBRs) and graphics-based messages (GBMs), which were all disseminated daily and weekly to participants in the intervention group via WhatsApp.

**Figure 1:**
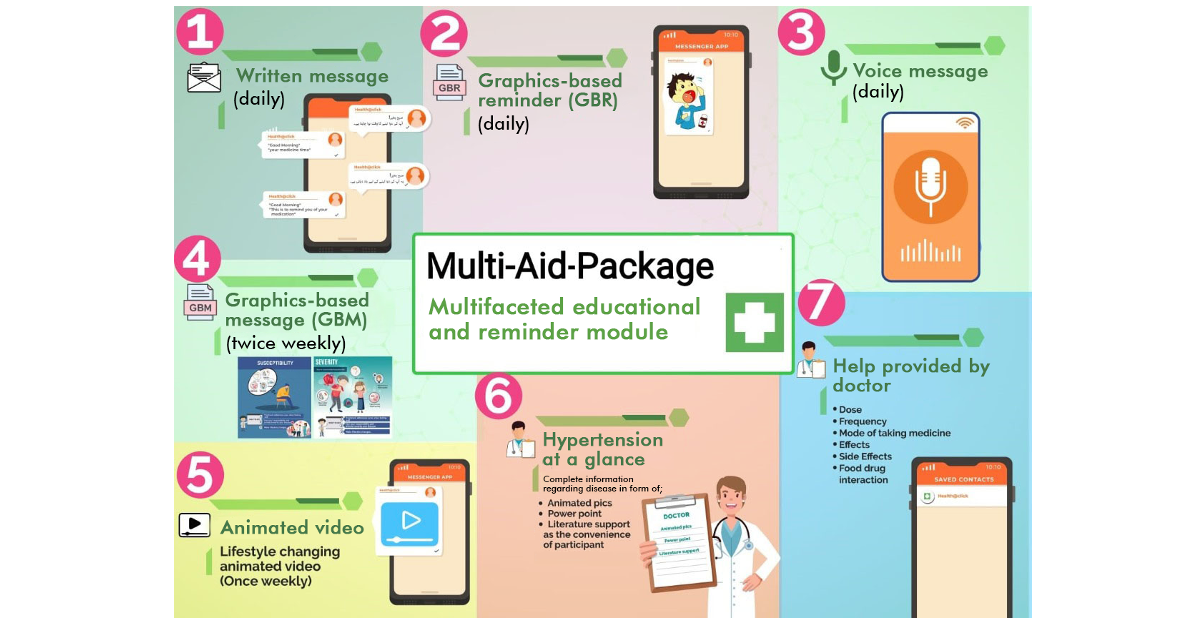
Contents of Multi-Aid-Package. AI: artificial intelligence.

Reminder text and voice messages for medication intake were in Urdu, as it was the most commonly used language among the study participants. Examples of such messages are “Good morning, it’s time for your medication” and “Good morning, this is a reminder for you to take your pills” (see items 1 and 3 in Figure 1).

GBMs were an animated series of messages developed with the help of a professional team of software developers (see item 4 in Figure 1). Furthermore, an animated video was also created by IT professionals with the help of clinical experts. The resulting animated video was divided into 3 sections: (1) awareness of hypertension, (2) the negative consequences of uncontrolled hypertension, and (3) medical and lifestyle changes for better health (see item 5 in Figure 1). In this module, participants were also provided with the “Hypertension at a Glance” component, a portfolio of instructional and educational tools that shows details of the condition’s causes, diagnosis, treatment, complications, and prognosis (see item 6 in Figure 1).

In addition to the components mentioned earlier, the module also contained live support provided by a certified doctor 24 hours a day. The support included information on the medicine’s dose, the dose frequency, the administration method, the effects of therapy on the present sickness, adverse effects, and interactions with particular meals (see item 7 in Figure 1). Live support and the “Hypertension at a Glance” portfolio were provided on a demand or need basis to only those participants who encountered problems from day 1 of the intervention, while the remaining components of Multi-Aid-Package were disseminated following the timetable provided. The contents of Multi-Aid-Package are summarized in Figure 1.

### Pilot Testing

In addition, the Multi-Aid-Package was subject to a pilot test among 44 patients with hypertension to determine if they could understand the module’s contents. As per usual hospital practice, only standard care was given to those in the control group. After being pilot tested, the intervention didn't change significantly. Only a few minor issues were seen, such as delivery issues, network issues, being outside of the coverage region, and unsuccessful file and video downloads. During the trial, this application’s final version was made available.

### Implementation of Multi-Aid-Package

**Figure 2:**
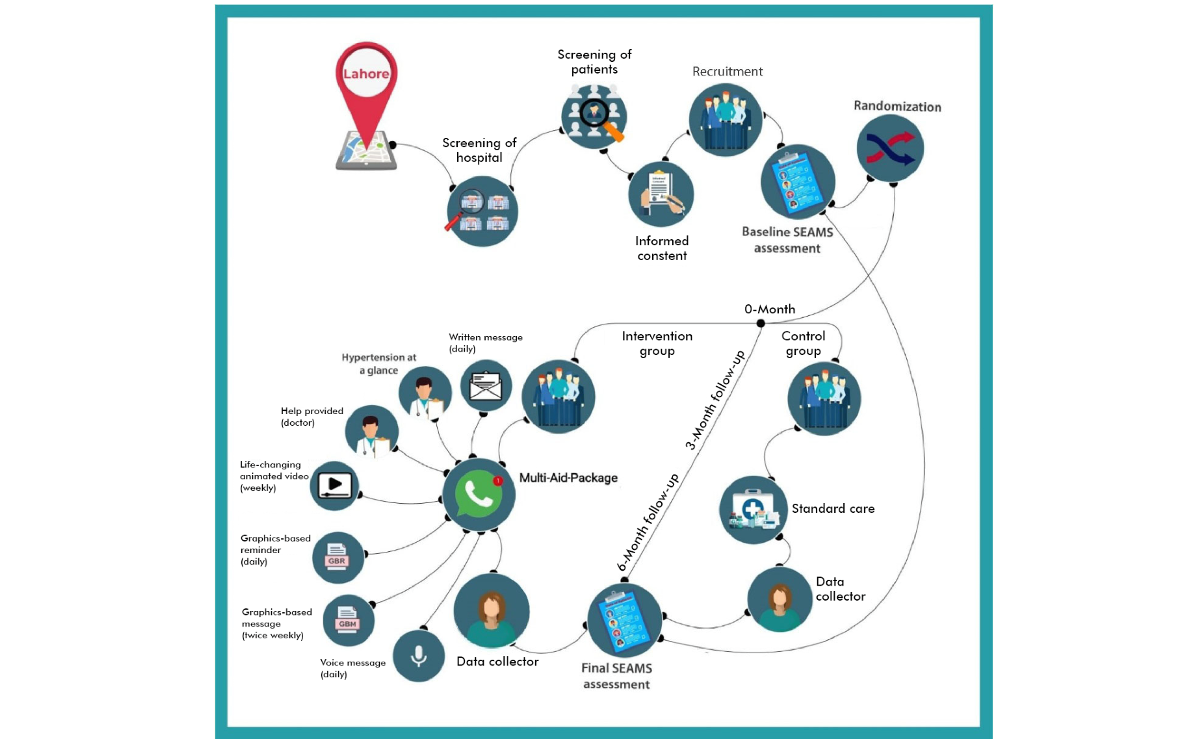
Trial flow. SEAMS: Self-Efficacy for Appropriate Medication Adherence Scale.

The trial’s design and execution adhered to the 2010 CONSORT criteria, which included a rigorous protocol for the provision of interventions to participants. This included the validation of the Multi-Aid-Package intervention in a pilot study, and an orientation and training session on the intervention was provided to the participants. Contact numbers were also provided to the participants in case they experienced any inconvenience. Moreover, a strict protocol was followed to disseminate the various contents of the multifaceted intervention. The implementation of Multi-Aid-Package was coordinated with the assistance of an IT specialist and 2 trained research assistants. In essence, these individuals were tasked to oversee the dissemination of the various contents of Multi-Aid-Package to the intervention group’s participants via WhatsApp and to support data collection. WhatsApp was used in this study because it contains a feature on its interface that indicates to the sender whether the receiver has seen the message: a sign on the message sent through this app changes its color to blue when the recipient sees the message. This was the only way to check whether the participants had read the message. Second, the outcome results illustrated whether the participants took their medication, as prescribed. Research staff members were trained and the intervention pretested to ensure quality control. Using a pre-established curriculum for training by experts, 2 days of on-site instruction sessions on hypertension and questionnaire completion and how to respond to typical queries related to medication adherence were provided to all recruited research workers. Finally, the intervention module was rolled out to the selected participants in the trial. Figure 2 shows the overall flow of the study implementation.

No financial or other benefits were provided to the participants, except 6 months of free-of-cost WhatsApp use. There were no other direct benefits provided.

### Participants’ Timeline

The recruitment process was completed from January to May 2021, and a total of 439 participants were included. From June to December 2021, the intervention group received the Multi-Aid-Package intervention. The intervention duration was 6 months. The 6-month time frame of the intervention was chosen based on data from the previous literature on the topic, which included studies conducted over 2 and 3 months [30,42], and the fact that 6 months is a reasonable time limit to observe changes in behavior.

### Data Collection

The data collection tool was a questionnaire in Urdu and English languages. The questionnaire was divided into 4 sections: A, B, C, and D.

Section A collected data on sociodemographic and health-related variables. Section B was the validated SEAMS questionnaire [34]. Section C collected self-reported pill-counting activity. Section D was a postintervention survey performed to assess the acceptability of the intervention. A 5-item Likert scale, with each item having 7 options, was used to evaluate participants’ perceptions of their intervention experience with regard to usefulness, simplicity of use, and fulfillment of information.

### Validity and Reliability of the Study Instrument

The internal consistency of SEAMS was good (Cronbach α=0.89). The test-retest reliability was moderate (Spearman coefficient=0.62, *P*<.001). The item total correlation coefficients ranged from 0.36 to 0.67, and the mean interitem correlation was 0.32 (range 0.08-0.71) [34].

The SEAMS questionnaire was translated from English to Urdu (SEAMS-U) in Pakistan using the standard “forward-backward” procedure. A convenient sample of 1011 patients with hypertension who were being treated at a tertiary care hospital in Lahore, Pakistan, was used to validate the translated version. The internal consistency of the translated questionnaire was good (Cronbach α=0.897). Cronbach α for part 1 was 0.838 and for part 2 was 0.789 using split-half reliability. The test-retest reliability was moderate (Spearman correlation=0.686, *P*<.001), and the intraclass correlation coefficient score was 0.814. The entire translation validity and reliability process was performed by our team and during publication.

### Data Management and Statistical Analysis

Data management was the responsibility of a study supervisor, a biostatistician, and 2 research assistants. First, the research assistants ensured that no data collection form was incomplete or missing. Next, the research supervisor received all the data in sealed boxes. If there were any missing pieces of information in the data, the participants were contacted via a phone call to finish the form. Lastly, a biostatistician entered, cleaned, and analyzed the data. SPSS version 26.0 (IBM Corp) and RStudio (version 4.0.3; Posit PBC) were used to analyze the data.

The intention-to-treat analysis was used in this study [43]. The Shapiro-Wilk test was performed to determine whether the data were normally distributed. Categorical data were represented using frequencies and percentages, while continuous data were represented using medians (IQRs). The nonparametric Mann-Whitney *U* test was used on the data for the primary and secondary outcomes between groups, while the Wilcoxon signed rank test was used for within-group differences between baseline and 6 months. For categorical variables, the chi-square test was used. The significance test was run with a *P* value of <.05. In addition, missing data were reported and treated using the single imputation approach. A generalized estimating equation (GEE) was used to control the covariates associated with the probability of affecting adherence to antihypertensive medication and the covariates that were significantly different between the intervention and control groups.

### Adverse Events

No other adverse results were reported, except the primary and secondary outcomes that were reported in relation to the participants and our intervention strategy. All COVID-19 standard operating procedures were strictly followed during recruitment, randomization, and data collection. Furthermore, no issues were reported regarding COVID-19.

### Ethical Considerations

The University Putra Malaysia (UPM) Ethical Committee on Human Research approved the research protocol (reference number: JKEUPM-2020-391) and the Institutional Review Board of the Sheikh Zayed Medical Complex Lahore (SZMC/IRB/163/2021). Participation in this trial was discretionary, and informed consent was obtained in writing from each participant before the start of the study. Strict confidentiality and privacy were ensured by assigning each participant an identification number to protect their identity [44]. The confidentiality of participant data was also ensured. This study was designed according to Good Clinical Practice (GCP) [45,46].

The study was designed to barely cause any risk to both patients and medical personnel. The participants were questioned in a private room, away from onlookers, in order to prevent minor psychological discomforts related to personal issues involving their income and the embarrassment that they might experience when answering questions about their subpar adherence status.

## Results

### Response Rate

**Figure 3:**
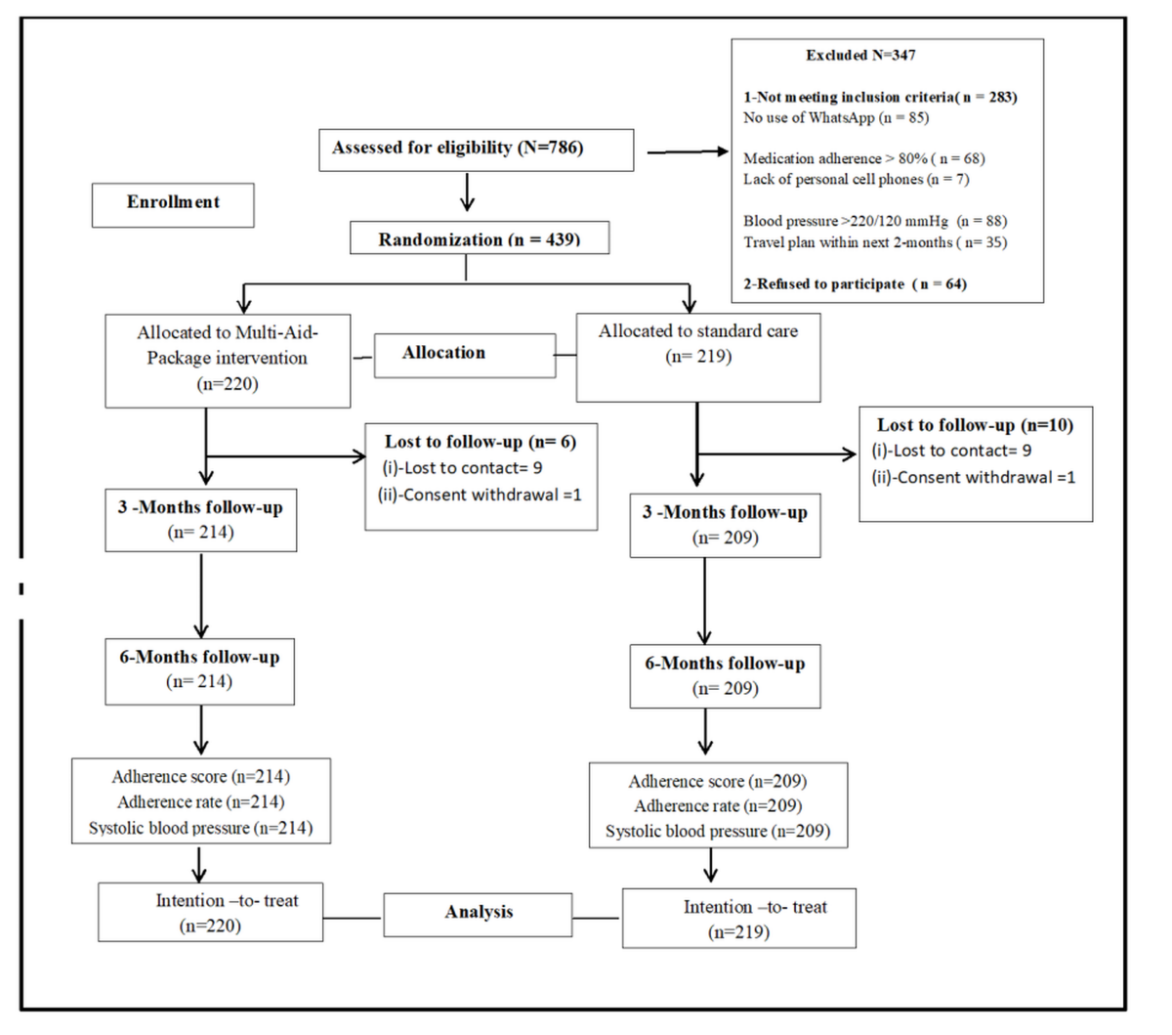
CONSORT flow diagram. CONSORT: Consolidated Standards of Reporting Trails.

From January to May 2021, a total of 786 participants were initially assessed based on the eligibility criteria. Of these, 347 (44.1%) participants were excluded based on inclusion criteria (n=283, 81.6%) and refusal to participate (n=64, 18.4%) in the trial. The details of the subcategories under “not meeting inclusion criteria” are elaborated in Figure 3. Of them, in June 2021, 439 participants fulfilled the criteria, consented to participate, and were randomly assigned to either the control group, which received standard care (n=219), or the intervention group, which received the Multi-Aid-Package (n=220). The randomization was performed according to the CONSORT flow diagram [31,47] (Figure 3). The total response rate at the end of the intervention was 423 (96.3%), with 209 (95.4%) participants in the control group and 214 (97.2%) participants in the intervention group until the completion of follow-up in December 2021. In the control group, 10 (4.8%) participants were lost to follow-up, while 1 (0.5%) participant withdrew consent. In the intervention group, 6 (2.8%) participants were lost to follow-up. The reason for the failure to follow up was loss of contact. No mortality was reported.

### Baseline Demographic Characteristics

At baseline, the 2 groups did not differ statistically significantly from each other across most of the variables, except gender, which had substantial differences among both groups (*P*=.02). In general, the intervention group’s baseline characteristics were similar to those of the control group regarding age, ethnicity, marital status, education, family status, employment, and monthly income. Most of the participants were aged between 30 and 49 years old and were male, with a graduate level of education and a high-income status (Table 1).

**Table 1:** Baseline characteristics of study participants according to their group allocation (N=439).^a^

Characteristics			Intervention group (n=220), n (%)		Control group (n=219), n (%)		*P* value^a^	
**Age (years)**								.30
	≤50	91 (41.4)		102 (46.6)				
	30-49	116 (52.7)		105 (47.9)				
	18-29	13 (5.9)		12 (5.5)				
**Gender**								.02
	Female	91 (41.4)		80 (36.5)				
	Male	129 (58.6)		139 (63.5)				
**Ethnicity**								.54
	Urdu	27 (12.3)		17 (7.8)				
	Punjabi	143 (65.0)		163 (74.4)				
	Suraiki	46 (20.9)		37 (16.9)				
	Others	4 (1.8)		2 (0.9)				
**Marital status**								.76
	Married	167 (75.9)		163 (74.4)				
	Single	32 (14.5)		25 (11.4)				
	Others	21 (9.5)		31 (14.2)				
**Education**								.59
	Primary and secondary	72 (32.7)		60 (27.4)				
	Graduate	80 (36.4)		71 (32.4)				
	Postgraduate	68 (30.9)		88 (40.2)				
**Do you smoke?**								.29
	No	171 (77.7)		151 (68.9)				
	Yes	44 (20.0)		57 (26.0)				
	Ex-smoker	5 (2.3)		11 (5.0)				
**Family status**								.31
	Joint family	125 (56.8)		126 (57.5)				
	Nuclear family	95 (43.2)		93 (42.5)				
**Employment**								.83
	Yes	192 (87.3)		182 (83.1)				
	No	28 (12.7)		37 (16.9)				
**Monthly income (PKR^b^)**								.60
	<10,000 (<US $35.96)^c^	1 (0.5)		1 (0.5)				
	10,000-25,999 (US $35.96-$93.49)	44 (20.0)		35 (16.0)				
	26,000-50,999 (US $93.50-$183.39)	31 (14.1)		30 (13.7)				
	51,000-100,000 (US $183.40-$359.60)	66 (30.0)		66 (30.1)				
	>100,000 (>US $359.60)	78 (35.5)		87 (39.7)				
**Use of reminder alarm**								.27
	Yes	42 (19.1)		67 (30.6)				
	No	178 (80.9)		152 (69.4)				

^a^*P*<.05 was considered statistically significant.

^b^PKR: Pakistani Rupee.

^c^An exchange rate of PKR 1=US $0.0036 was used.

### Baseline Health-Related Characteristics

Regarding health-related characteristics, there was no significantly significant difference between the 2 groups. Additionally, the median SEAMS score of the control group was substantially higher than that of the intervention group (*P*=.03). Otherwise, the 2 groups had a balanced distribution of subjects regarding the duration of hypertension, comorbid conditions, the number of medications used daily, dose frequency, SBP, and controlled status of SBP<140 mmHg (Table 2).

**Table 2:** Comparison of health-related characteristics between intervention and control groups (N=439).^a^

Characteristics			Intervention group (n=220)		Control group (n=219)		*P* value	
**Duration of hypertension (years), n (%)**								.40
	<1	22 (10.0)		22 (10.0)				
	1-5	79 (35.9)		82 (37.4)				
	>5	119 (54.1)		115 (52.5)				
**Concomitant disease, n (%)**								.34
	Yes	141 (64.1)		142 (64.8)				
	No	79 (35.9)		77 (35.2)				
**Comorbid conditions, n (%)**								.57
	1	87 (39.5)		88 (40.2)				
	>1	133 (60.5)		131 (59.8)				
**Daily medication number, n (%)**								.47
	<5	126 (57.3)		117 (53.4)				
	5-9	74 (33.6)		83 (37.9)				
	>10	20 (9.1)		19 (8.7)				
**Daily dose frequency, n (%)**								.91
	Once daily	64 (29.1)		55 (25.1)				
	Twice daily	104 (47.3)		111 (50.7)				
	Thrice daily	52 (23.6)		53 (24.2)				
**Controlled SBP^b^ <140 mmHg, n (%)**								.72
	Uncontrolled	217 (98.6)		215 (98.2)				
	Controlled	3 (1.36)		4 (1.82)				
**Pill counting, n (%)**								—^c^
	Nonadherent	220 (100.0)		219 (100.0)				
	Adherent	0		0				
SBP, median (IQR)			159 (23)		159 (27)		.77	
SEAMS^d^ adherence score, median (IQR)			19.5 (5)		21.0 (6)		.03	

^a^*P*<.05 was considered statistically significant.

^b^SBP: systolic blood pressure.

^c^Not applicable.

^d^SEAMS: Self-Efficacy for Appropriate Medication Adherence Scale.

### Effect of the Multi-Aid-Package Intervention on Medication Adherence

The effect of the Multi-Aid-Package intervention on medication adherence on the 2 groups at baseline and 6 months was measured using the median (IQR) SEAMS score. At baseline, the median SEAMS score was 19.5 (IQR 5) for the intervention group and 21 (IQR 6) for the control group. Compared to the intervention group, the control group’s median SEAMS score was significantly higher (1.5 points; *P*=.011). At 6-month follow-up, the median SEAMS score was significantly different between the intervention and control groups (*P*<.001).

Regarding the medication adherence status, at baseline, all participants were nonadherent; therefore, no further analysis could be performed. At 6 months, however, there was an increase in adherent patients between the intervention and control groups (difference n=81, 18.4% of patients; *P*<.001; Table 3).

The effect of the Multi-Aid-Package intervention on medication adherence within groups at baseline and 6 months was also measured using the median (IQR) SEAMS score. At baseline, the median SEAMS score within the intervention group was 19.5 (IQR 5), which increased to 32 (IQR 11) at the 6-month follow-up, with a median difference of 12.5 points. The median SEAMS score statistically significantly changed from baseline to 6 months (*P*<.001), while there was no statistically significant change in the median SEAMS score in the control group from baseline to 6 months (*P*=.29). A total of 83 (37.7%) participants achieved adherent status in the intervention group (*P*<.001), while 2 (0.9%) participants achieved adherent status in the control group (*P*=.78) from baseline to 6 months (refer to Multimedia Appendix 1).

**Table 3:** Primary outcomes for the intervention and control groups from baseline to 6 months (N=439).

Variable	Intervention group (n=220)	Control group (n=219)	Difference (intervention – control)	Test statistics	*P* value
SEAMS^a^ score at baseline, median (IQR)	19.5 (5)	21.0 (6)	–1.5	*U*^b^=20,717.500	.01
SEAMS score at 6 months, median (IQR)	32.0 (11)	21.0 (6)	11	*U*=10,490.000	<.001
Adherence status at 6 months, n (%)	83 (37.72)	2 (.91)	81	95.266^c^	<.001

^a^SEAMS: Self-Efficacy for Appropriate Medication Adherence Scale.

^b^Mann-Whitney *U* test.

^c^Fisher exact test.

### Effect of the Multi-Aid-Package Intervention on the SBP

At baseline, there was no difference in the SBP between the 2 groups. At 6 months, however, the median SBP was statistically different between the intervention and control groups (*P*<.001). A binary variable “controlled systolic blood pressure” was computed to evaluate the success of the treatment. The controlled SBP code was “Controlled <140 mmHg and uncontrolled >140 mmHg.”

Overall, at 6 months, the number of patients with uncontrolled hypertension decreased by 46 in the intervention group (*P*<.001) but remained unchanged in the control group (*P*=.724; Table 4).

At baseline, the median SBP in the intervention group was 159 (IQR 23) mmHg, which decreased to 155 (IQR 29) mmHg, with a median difference of 4 mmHg. The median SBP significantly changed from baseline to 6 months (*P*<.001), while there was no statistically significant change in the median SBP in the control group from baseline to 6 months (*P*=.31). A total of 49 (22.3%) participants achieved controlled SBP status in the intervention group (*P*<.001), whereas there was no change in the control group’s controlled SBP status (*P*=.78) from baseline to 6 months (refer to Multimedia Appendix 1).

**Table 4:** Secondary outcome change between intervention and control groups from baseline to 6 months (N=439).

Variable	Intervention group (n=220)	Control group (n=219)	Difference (intervention – control)	Test statistics	*P* value
SBP^a^ (mmHg) at baseline, median (IQR)	159 (23)	159 (27)	0	*U*^b^=23,768.000	.81
SBP (mmHg) at 6 months, median (IQR)	155 (29)	162 (17)	–7	*U*=18,276.000	<.001^c^
Controlled SBP (mmHg) at baseline, n (%)	3 (1.36)	4 (1.83)	–1	N/A^d^	.72
Controlled SBP (mmHg) at 6 months, n (%)	49 (22.27)	4 (1.83)	45	43.221^e^	<.001^c^

^a^SBP: systolic blood pressure.

^b^Mann-Whitney *U* test.

^c^Significant *P* value.

^d^Not applicable.

^e^Fisher exact test.

### Covariates Affecting Medication Adherence

A GEE was used to control the covariates associated with the probability of affecting adherence to antihypertensive medication using pill counting and the covariates significantly different between the intervention and control groups. We used the forward method. A working correlation matrix was gender. A total of 3 factors were found significant: group, time, and age. The group variable significantly contributed to medication adherence. The intervention group had a 1.714 times higher probability of being adherent to antihypertensive medication than the control group (adjusted odds ratio [AOR] 1.714, 95% CI 2.387-3.825; *P*<.001). Time points also contributed significantly to medication adherence. A 6-month postintervention time had a 1.837 times higher probability of showing adherence to antihypertensive medication than baseline (AOR 1.837, 95% CI 1.625-2.754; *P*<.001). Age also contributed significantly. The 18-29 years of age group was found more likely to be adherent to antihypertensive treatment, with a 1.618 times higher probability than the other 2 age groups (AOR 1.618, 95% CI 0.225-1.699; *P*<.001). Income was a significant predictor for adherence to antihypertensive treatment (Table 5).

**Table 5:** Effect of the Multi-Aid-Package intervention on medication adherence, with adjusted covariates by the GEE^a^ (N=439).

Variable		B^b^ (SE)	Wald chi-square (*df*=1)	AOR^c^, exp(B) (95% CI)	*P* value
**Group**					
	Intervention	0.672 (0.863)	4.813	1.714 (2.387-3.825)	<.001^d^
	Control	Reference	—^e^	—	—
**Time point**					
	6 months	0.748 (0.216)	2.765	1.837 (1.625-2.754)	<.001^d^
	Baseline	Reference	—	—	—
**Age (years)**					
	≥50	–4.302 (0.574)	2.953	0.014 (0.002-0.074)	<.001^d^
	>18	Reference	—	—	—
**Gender**					
	Female	0.141 (0.287)	0.242	1.152 (0.655-2.025)	.62
	Male	Reference	—	—	—
**Education**					
	Primary and secondary	0.583 (0.365)	2.548	1.792 (0.876-3.667)	.11
	Graduate	0.207 (0.358)	0.332	1.230 (0.609-2.485)	.56
	Postgraduate	Reference	—	—	—
**Monthly income (PKR^f^)**					
	26,000-50,999 (US $93.50-$183.39)^g^	–0.441 (0.379)	1.346	0.644 (0.306-1.355)	.25
	51,000-100,000 (US $183.40-$359.60)	–0.933 (0.672)	0.936	0.393 (0.157-0.983)	.17
	>100,000 (>US $359.60)	Reference	—	—	—
**Duration of hypertension (years)**					
	<1	–0.194 (0.447)	0.189	0.823 (0.343-1.978)	.66
	1-5	–0.011 (0.300)	0.001	0.989 (0.549-1.780)	.97
	>5	Reference	—	—	—
**Concomitant disease**					
	Yes	–0.197 (0.286)	0.472	0.821 (0.468-1.441)	.49
	No	Reference	—	—	—
**Comorbid conditions**					
	1	0.043 (0.399)	0.012	1.044 (0.478-2.282)	.91
	>1	Reference	—	—	—
**Daily medication number**					
	<5	0.357 (0.829)	0.186	1.429 (0.281-7.259)	.67
	5-9	–0.148 (0.686)	0.046	0.863 (0.225-3.313)	.83
	>10	Reference	—	—	—
**Daily dose frequency**					
	1	0.245 (0.680)	0.129	1.277 (0.337-4.848)	.72
	2	0.041 (0.566)	0.005	1.042 (0.343-3.163)	.94
	3	Reference	—	—	—

^a^GEE: generalized estimating equation.

^b^B: unstandardized β.

^c^AOR: adjusted odds ratio.

^d^*P*<.05 was considered statistically significant.

^e^Not applicable.

^f^PKR: Pakistani Rupee.

^g^An exchange rate of PKR 1=US $0.0036 was used.

### Technology Acceptance Feedback

At the end of the study, an intervention acceptance survey for Multi-Aid-Package was performed. A total of 214 (97.3%) participants from the intervention group participated in the survey. The survey consisted of 5 questions on (1) the information provided by Multi-Aid-Package about the disease, disease management, and complications (1 question); (2) how easy the participants found the intervention to use (2 questions); and (3) utility (2 questions). Each question offered 7 possible answers. Ratings ranged from 7 to 35. The minimum score was 7, while the maximum score was 35. Next, the mean score was calculated for the 214 (97.3%) participants. The Multi-Aid-Package intervention received a mean score of 33.21 (SD 4.39) of 35 points (94.8%), with good feedback on its usefulness, simplicity of use, and fulfillment of information for treating hypertension.

## Discussion

### Principal Findings

The comprehensive and unique multifaceted Multi-Aid-Package comprised 7 potential components, including continuous reminders integrated with education and support components. Multi-Aid-Package was designed for the intervention group and revealed a significant increase in adherence to antihypertensive medication and a substantial reduction in SBP in patients with hypertension. This study showed that using Multi-Aid-Package led to a significant improvement in medication adherence among patients who were nonadherent to their antihypertensive medication at the beginning of the trial.

Similar results from an existing body of literature concur with this trial’s findings, where SMS text message interventions revealed positive results in patients with hypertension compared to controls [17,29]. Some trials have also shown significant results in patients with hypertension using advanced cell phone apps [48-50]. Overall, mHealth technology interventions have revealed positive results in patients with CVD [51]. In another trial, an SMS text message intervention demonstrated substantial improvement in adherence to treatment, from 49% to 62.3%, in patients with hypertension [52]. In the previous literature, mHealth interventions have been reported to lower blood pressure and improve medication adherence, with adequate acceptance and feasibility [16,23,53-56]. Patients also significantly benefit over time with self-management and blood pressure control [57-60]. However, a few trials were unable to reveal any significant improvement in medication adherence post intervention: one used a mobile app, while the other studies used a web-based talking intervention to enhance medication adherence in CVD [16,30]. Similarly, another trial using mailing and automated calls [61] and one using video interventions in patients with stroke [62] were unable to reveal any substantial change. mHealth is also paramount in medication adherence in other chronic illnesses, such as tuberculosis, CVD, diabetes, and chronic liver diseases [63-66].

### Research Innovation and Clinical Implications

Multi-Aid-Package is a modified version of the preexisting literature on this subject as it contains multiple facets (SMS text messages, apps, interactive messages, and calls) in 1 application compared to only 1 or 2 facets per app in other interventions. Previous trials have used different facets of mHealth, for instance, SMS text messaging interventions used to improve adherence to antihypertensive medication [29,67], interactive voice interventions [68], talking treatment interventions [30], advanced mobile apps [48,50], and mail-outs [61]. Some of these interventions have demonstrated positive results, while others have been unable to reveal any improvement or insignificant improvement in adherence to antihypertensive medication. The unique aspect of this study is that the Multi-Aid-Package intervention combines multiple facets in 1 intervention. Multi-Aid-Package is superior because it contains 7 different parts, such as SMS text messaging, videos, and graphics.

Multi-Aid-Package is a comprehensive and effective tool for enhancing antihypertensive medication adherence and subsequently managing the SBP. Much preexisting literature supports our findings. In most studies, intervention group participants have reported being adherent more likely compared to the controls, where the intervention eventually altered health beliefs concerning medication adherence but was unable to show any significant effect on the SBP. For example, a cell phone app for patients with hypertension improved medication adherence but failed to significantly control the SBP in an intervention arm compared to the control arm [48]. Similarly, another 12-month trial found a minor change in the SBP [29] compared to our study, which revealed better results, even with a shorter duration. mHealth interventions are also influential in changing lifestyles [67]. Although there can be various explanations for no significant or even a minute change in the SBP despite considerable improvement in medication adherence, factual evidence shows that a reasonably long time is needed to see a change in clinical outcomes. Nevertheless, becoming highly adherent to therapy is essential. The literature also emphasizes that to obtain more clinical benefits, patients must strictly adhere to their antihypertensive medications [69]. There is no evidence of the efficiency of interventions in lowering the DBP, although an increased SBP is the primary aim of antihypertensive medication. Furthermore, according to epidemiological research, untreated hypertension, especially the SBP, ought to be the main goal for hypertension treatment [70].

It is crucial to emphasize that using Multi-Aid-Package in the framework of clinical care in a resource-limited setting can alone help support patients in managing their hypertension. Several studies have found that improving medication adherence has a higher impact on clinical outcomes linked to additional assistance, primarily through connections to health care professionals [71]. Some effective interventions has shown that support via a cell phone can be provided without further blood pressure monitoring [72] and with nonadherent patients not being contacted by any health care providers [73]. In our study, there was no permanent connection between the patient, the health care provider, and the targeted blood pressure monitor.

Conclusively, to the best of our knowledge, this multifaceted approach is the first technology-based intervention in Pakistan to be built comprehensively and uniquely using only WhatsApp, which was cost-, time-, and resource-efficient. In contrast to prior cell phone–based interventions, this multifaceted intervention has a more substantial impact on antihypertensive medication adherence and SBP outcomes.

### Future Suggestions

Further exploration by covering multiple cities, a large sample size, and a longer duration of follow-up is required to validate the results of this study. Our findings cannot be generalized to populations with different sociodemographic and medical profiles, so more diverse studies are required to generalize the findings to a wider population. There are recommendations for more sophisticated designs and efficient interventions to enhance medication adherence in CVD [21]. To improve outcomes, we suggest implementing new educational interventions with more effective designs and sophisticated adherence measurement techniques (mobile apps or devices) at a relatively low cost and implementing successful treatments in clinical settings. A recent study compared an innovative mHealth strategy to peer counseling to improve adherence to medication in patients with hypertension [51]. Future trials should consider the type of antihypertensive medication being taken. Finally, high-quality research is needed to explore mixed qualitative evidence with quantitative studies.

### Strengths and Limitations

Two methods were used to measure medication adherence to strengthen the method of measurement and to obtain robust findings, supported by a preexisting body of literature on the subject [74]. The SBP was also assessed as a secondary outcome to increase the credibility of adherence to medication in patients with hypertension. An interim analysis was performed to monitor the dropout status, increasing the trial’s strength. All the steps in the trial were in line with recent SPIRIT guidelines [75]. The Multi-Aid-Package intervention could help minimize inequity and prevent discrimination among different sociodemographic groups. Technology acceptance feedback was also assessed at the study’s end, and the intervention received excellent feedback.

This trial also has a few limitations. First, the trial was a single-center study conducted in only 1 city due to time constraints, limited funds, and response burden. Therefore, extension over the entire province or multiple towns might be impossible. The possible effect size at various sites may vary, which may potentially affect the findings and may constrain external validity. In addition, the study continued for 6 months; consequently, the researchers might not be able to ascertain the effect of adherence to medication on treatment outcomes for a longer duration. The study also did not consider the difference in gender between groups, and this might be a good area for future research. Finally, self-reporting was used to assess medication adherence. Social desirability bias could cause self-report questionnaires to overestimate genuine adherence [76]. However, several technology-based or mHealth methods exist to follow and measure adherence, which could not be used in the study due to the nonavailability of such devices, poor communication of patients, or fear of loss to follow-up. Please refer to the eHealth CONSORT checklist for more information about this Multi-Aid-Package trial [77].

### Conclusions

In the context of this study, the multifaceted Multi-Aid-Package is an effective mHealth intervention that increased medication adherence among patients with hypertension and subsequently improves their SBP readings. The findings revealed a statistically significant change in the medication adherence score and pill-counting rates and a reduction in the SBP 6 months after the intervention began. The outcomes also demonstrated that users value Multi-Aid-Package for its applicability, ease of use, and informational content for the management of hypertension. Multi-Aid-Package should be considered as an approach for boosting the adherence of hypertension patients to their medication in Pakistan and other similar LMICs.

## Appendix

Multimedia Appendix 1 Primary and secondary outcomes for the intervention and control groups.

Multimedia Appendix 2 CONSORT-eHEALTH checklist (V 1.6.1).

## Abbreviations

AOR: adjusted odds ratio
CONSORT: Consolidated Standards of Reporting Trails
CVD: cardiovascular disease
DBP: diastolic blood pressure
GBM: graphics-based message
GBR: graphics-based reminder
GEE: generalized estimating equation
LMIC: low- and middle-income country
mHealth: mobile health
PKR: Pakistani Rupee
SBP: systolic blood pressure
SEAMS: Self-Efficacy for Appropriate Medication Adherence Scale
