# Supplementary material for: Effectiveness of a Multifaceted Mobile Health Intervention (Multi-Aid-Package) in Medication Adherence and Treatment Outcomes Among Patients With Hypertension in a Low- to Middle-Income Country: Randomized Controlled Trial
Source: JMIR Mhealth Uhealth. 2024 Jun 19;12:e50248. doi: 10.2196/50248 (PMC11222770; doi:10.2196/50248)
Supplement: Multimedia Appendix 1 [file mhealth_v12i1e50248_app1.pdf]

Appendix

Appendix 1

Primary outcome (Medication adherence) within intervention and control groups from baseline to 6 months

Table 4. Primary outcome within intervention and control groups from baseline to 6 months

Groups	Time points (Time 0 vs. Time 2)				*P* value
	Baseline n (%)	6 months n (%)	Difference	Test statistics	
**Intervention group SEAMS Score Median(IQR)**	19.50 (5)	32.00 (11)	12.5	-10.924[b]	< 0.001*
**Control group SEAMS Score Median(IQR)**	21.00 (6)	21.00 (6)	0	-1.058[b]	0.290
**Intervention group Adherence status**	0	83 (37.72)	83 (37.72)	82.266[a]	< 0.001*
**Control group Adherence status**	0	2 (0.91)	2 (0.91)	N/A	0.782

[a]: chi-square
[b]: Wilcoxon Signed-rank test

Secondary outcome(SBP) within intervention and control groups from baseline to 6 months

Table 6. Secondary outcome within intervention and control groups from baseline to 6 months

Group	Time points				*P* value
	Baseline	6 months	Difference	Test statistics	
**Intervention group SBP Median(IQR) mmHg**	159 (23)	155 (29)	-4	-7.211[b]	< 0.001*
**Control group SBP Median(IQR) mmHg**	159 (27)	162 (17)	3	-1.007[b]	0.314
**Intervention group Controlled SBP No. (%) mmHg**	3 (1.36)	49 (22.27)	46 (-20.90)	59.683[a]	< 0.001*
**Control group Controlled SBP No. (%) mmHg**	4 (1.82)	4 (1.82)	0	N/A	0.782

[a:] chi-square test
[b]: Wilcoxon Signed-rank test
